# First-trimester plasma asprosin and its association with gestational diabetes mellitus: A prospective cohort study

**DOI:** 10.1371/journal.pone.0359945

**Published:** 2026-10-22

**Authors:** Sathaphone Inthavong, Phudit Jatavan, Sirinart Kumfu, Nipon Chattipakorn, Theera Tongsong

**Affiliations:** 1 Department of Obstetrics and Gynecology; Faculty of Medicine, Chiang Mai University, Chiang Mai, Thailand; 2 Fetal Center, Faculty of Medicine, Chiang Mai University, Chiang Mai, Thailand; 3 Department of Physiology, Faculty of Medicine, Chiang Mai University, Chiang Mai, Thailand; 4 Center of Excellence in Cardiac Electrophysiology Research, Chiang Mai University, Chiang Mai, Thailand; West Virginia University, UNITED STATES OF AMERICA

## Abstract

**Background:**

Asprosin a glucogenic adipokine associated with insulin resistance, has been found to be elevated in the plasma of women with gestational diabetes mellitus (GDM). Emerging evidence indicates that it can serve as a promising biomarker for the diagnosis of GDM during the second trimester. However, its utility in the first trimester has not yet been investigated. Therefore, the present study aimed to establish first-trimester reference ranges for plasma asprosin and to evaluate its predictive performance for the subsequent development of GDM.

**Methods:**

In this prospective cohort study, low-risk women with singleton pregnancies at 10–14 weeks of gestation were consecutively enrolled. Participants with pregestational diabetes were excluded. Baseline clinical characteristics were documented, and blood samples were obtained for the measurement of plasma asprosin concentrations. The diagnosis of GDM at 24–28 weeks of gestation was established using the two-step screening approach.

**Results:**

Among the 302 participants, 98 (32.5%) developed GDM. Asprosin concentrations did not differ significantly across gestational ages between 10 and 14 weeks. However, asprosin levels increased significantly with maternal BMI (p < 0.001), demonstrating a quadratic relationship. A first-trimester reference range for plasma asprosin was established. Nevertheless, first-trimester asprosin showed poor predictive performance for subsequent GDM (AUC = 0.516), whereas maternal BMI and age showed modest predictive ability.

**Conclusion:**

First, a reference range for first-trimester asprosin was established, which may serve as a valuable benchmark for future investigations examining various aspects of diabetes in pregnancy. Second, first-trimester asprosin concentrations did not discriminate between women who subsequently developed GDM and those who did not.

## Introduction

Gestational diabetes mellitus (GDM), defined as glucose intolerance first recognized during pregnancy [1], is a common obstetric complication associated with adverse maternal and neonatal outcomes, including macrosomia, preeclampsia, preterm birth, and increased long-term risks of type 2 diabetes mellitus (DM) [2,3]. Current screening relies on oral glucose tolerance test (OGTT) at a 24–28 week [4], which may be too late to prevent early metabolic exposure. Although first-trimester risk stratification has been explored, models based solely on maternal clinical factors show limited predictive performance, highlighting the need for more robust biomarkers. Asprosin has recently emerged as a potential biomarker for GDM and may help stratify women into high- and low-risk groups. If shown to reliably predict GDM, asprosin could serve as a valuable first-trimester marker for identifying women at increased risk.

Asprosin, a fasting-induced glucogenic adipokine encoded by the *FBN1* gene, is primarily secreted by white adipose tissue. It stimulates hepatic glucose release via the G protein–cAMP-PKA pathway [5] and crosses the blood-brain barrier to activate AgRP neurons, thereby increasing appetite [6]. Emerging evidence indicates that asprosin influences the hypothalamus-pituitary-gonadal axis, a key neuroendocrine system regulating mammalian reproduction [7]. Recent meta-analysis has shown that asprosin is significantly higher in patients with either type 2 DM, metabolic syndrome, or obesity, compared with controls [8]. Several studies report higher asprosin levels in women with GDM, particularly in late gestation [9–11]. Zhong et al. [10] demonstrated elevated asprosin levels at 18–20 weeks in women who later developed GDM, suggesting potential for early detection. Elevated maternal and cord blood levels have also been associated with GDM, and macrosomia. Boz et al. [11] reported that a second-trimester cutoff >31.7 ng/mL yielded 93.3% sensitivity and 90.9% specificity for GDM detection. However, evidence regarding first-trimester predictive value remains limited.

Emerging evidence suggests that asprosin is a promising novel biomarker closely associated with GDM and type 2 DM. This raises the possibility that asprosin could serve as an early screening marker for subsequent GDM and potentially aid in identifying pregestational diabetes at the initial antenatal visit. Because women who later develop GDM often exhibit subclinical metabolic dysfunction that becomes clinically evident as insulin resistance increases in mid-pregnancy, elevated asprosin levels in early gestation may reflect underlying insulin resistance. We therefore hypothesized that first-trimester asprosin concentrations are higher in women who subsequently develop GDM and may predict later disease onset.

To date, no reliable first-trimester biomarker has been established for predicting GDM. Although asprosin has shown promise in mid-pregnancy, its predictive value in early gestation remains uncertain. Establishing normal reference ranges in the first trimester is essential before clinical implementation. Accordingly, this study aimed to define first-trimester reference ranges for asprosin and to evaluate its performance in predicting the subsequent development of GDM.

## Materials and methods

This prospective cohort study was conducted at the antenatal care clinic of Chiang Mai University Hospital, a tertiary referral center, between 06 January 2025 and 30 November 2025. The study protocol was approved by the Institutional Review Board, Faculty of Medicine, Chiang Mai University (Research Ethics Committee Panel 5; study code OBG-2567–0726). All participants were invited to join the study and provided written informed consent prior to enrollment, after receiving detailed information about the study procedures.

The study population was enrolled at our antenatal clinic through consecutive recruitment. The inclusion criteria were: (1) gestational age of 10–14 weeks based on a reliable last menstrual period and first-trimester crown-rump length measurement; (2) singleton pregnancy confirmed by first-trimester ultrasound; (3) maternal age between 20 and 45 years; and (4) low risk pregnancy (no underlying medical disease). Exclusion criteria included: (1) pre-existing diabetes mellitus diagnosed before pregnancy; (2) HbA1c ≥ 6.5% at enrollment; (3) current use of medications known to affect glucose metabolism; and (4) loss to follow-up or failure to undergo the OGTT at 24–28 weeks of gestation.

At the enrollment visit, trained research personnel conducted standardized interviews using structured case report forms to collect comprehensive demographic, obstetric, and medical history information. The specific variables extracted for analysis included maternal age, ethnicity, parity, history of GDM, first-degree family history of diabetes mellitus (DM), and personal medical history, specifically chronic hypertension, and dyslipidemia.

### Laboratory analysis

Maternal blood samples were collected at 10–14 weeks of gestation to measure plasma asprosin concentrations. Asprosin levels were quantified using the Human Asprosin SimpleStep ELISA® Kit (FBN1). The assay employs an affinity tag-labeled capture antibody and a reporter-conjugated detector antibody, which immunocapture the asprosin analyte in solution before immobilization onto the microplate. The signal is generated proportionally to the bound analyte and measured at 450 nm following tetramethylbenzidine (TMB) development. The calculated minimal detectable dose for the assay is 0.92 ng/mL, with an intra-assay precision coefficient of variation (CV) of 7.9%.

Between 24 and 28 weeks of gestation, participants underwent routine two-step screening for GDM. First, a 50-g oral glucose challenge test (GCT) was administered regardless of fasting status. A 1-hour plasma glucose value ≤140 mg/dL was considered normal, whereas a value ≥ 140 mg/dL prompted a follow-up 100-g oral glucose tolerance test (OGTT) within one week. Glucose levels were measured at fasting and at 1-, 2-, and 3-hour intervals, with cut-off values of ≥105 mg/dL, ≥ 190 mg/dL, ≥ 165 mg/dL, and ≥ 145 mg/dL, respectively. A diagnosis of GDM was made if at least two values met or exceeded these thresholds. If only the fasting glucose value was abnormal and all other OGTT values were within the normal range, the test was repeated one month later. If only one of the non-fasting values was abnormal, the result was considered normal and no diagnosis of GDM was made. This approach classified participants into two groups: the GDM group and the non-GDM group. Additional GDM screening after 28 weeks of gestation could be performed if clinical indicators, such as persistent glucosuria on dipstick testing were observed. Screening and diagnostic decisions were made, by attending physicians in the antenatal clinic. Participants diagnosed with GDM at any point were reclassified into the GDM group. All participants were followed up by the research team.

To adequately address the primary outcome, this study required a minimum sample size of 47 cases of GDM to achieve 80% statistical power at a 95% confidence level.

**Statistical analyses** were conducted using the Statistical Package for the Social Sciences (SPSS), version 26.0 (IBM Corp., 2019; IBM SPSS Statistics for Windows, Version 26.0, Armonk, NY, USA). Continuous variables were expressed as mean ± SD or median with interquartile range (IQR), as appropriate based on data distribution. Comparisons of baseline characteristics between the GDM and non-GDM groups were performed using the Student’s t-test or Mann-Whitney U test for continuous variables, as appropriate, and the chi-square test for categorical variables. The reference range of asprosin levels was determined and adjusted for potential confounding factors using a generalized linear model. The discriminatory ability of asprosin and other potential risk factors for predicting GDM was assessed using binary logistic regression and the area under the receiver operating characteristic (ROC) curve (AUC). A two-tailed p-value < 0.05 was considered statistically significant.

## Results

During the study period, 324 women attended the antenatal care clinic and were initially enrolled, as illustrated in Fig 1. Of these, 302 fulfilled the inclusion criteria and were subsequently included in the final analysis. GDM was diagnosed in 98 women (32.5%) at 24–28 weeks of gestation. Comparative analysis of baseline characteristics between women who developed GDM and those who did not is presented in Table 1. Women in the GDM group were significantly older than those in the non-GDM group (33.1 ± 4.4 vs. 30.7 ± 4.9 years, p < 0.001). Additionally, pre-pregnancy weight and pre-pregnancy BMI were significantly higher among women who developed GDM (24.9 ± 4.9 vs. 23.0 ± 4.3 kg/m^2^, p < 0.001). A first-degree family history of diabetes mellitus and a prior history of dyslipidemia were also significantly more prevalent in the GDM group. In contrast, no statistically significant differences were observed between the two groups with respect to parity or a history of chronic hypertension.

**Table 1:** Characteristics of GDM and non-GDM group.

Variables	Non-GDM group N: 204	GDM groupN: 98	P value
Maternal age (year)	30.7 + 4.9	33.1 + 4.4	< 0.001
Maternal pre-pregnancy BMI	23.0 + 4.3	24.9 + 4.9	< 0.001
Pre-pregnancy weight (kg)	57.7 + 12.5	61.9 + 13.4	0.007
Family history of DM	23 (11.3%)	23 (23.5%)	0.006
Chronic hypertension	2 (1.0%)	3 (3.1%)	0.185
Dyslipidemia	0 (0.0%)	4 (4.1%)	0.004
Parity			0.868
Nulliparous	129 (63.2%)	61 (62.9%	
Parous	75 (36.8%)	37 (37.8%)	
Gestational age at recruitment	12.3 + 0.9	12.3 + 1.0	0.590
Gestational age at GDM screening	24.8 + 1.9	24.8 + 1.4	0.905
Plasma asprosin (ng/mL), mean+SD	59.4 + 20.0	59.7 + 19.0	0.887
Plasma asprosin (ng/mL), median (IQR)	57.9 (45.8-69.3)	56.4 (46.8-71.8)	0.652^#^

# Mann-Whitney-U test.

GDM, gestational diabetes mellitus; BMI, body mass index; DM, diabetes mellitus; SD, standard deviation; IQR, interquartile range.

**Fig 1:**
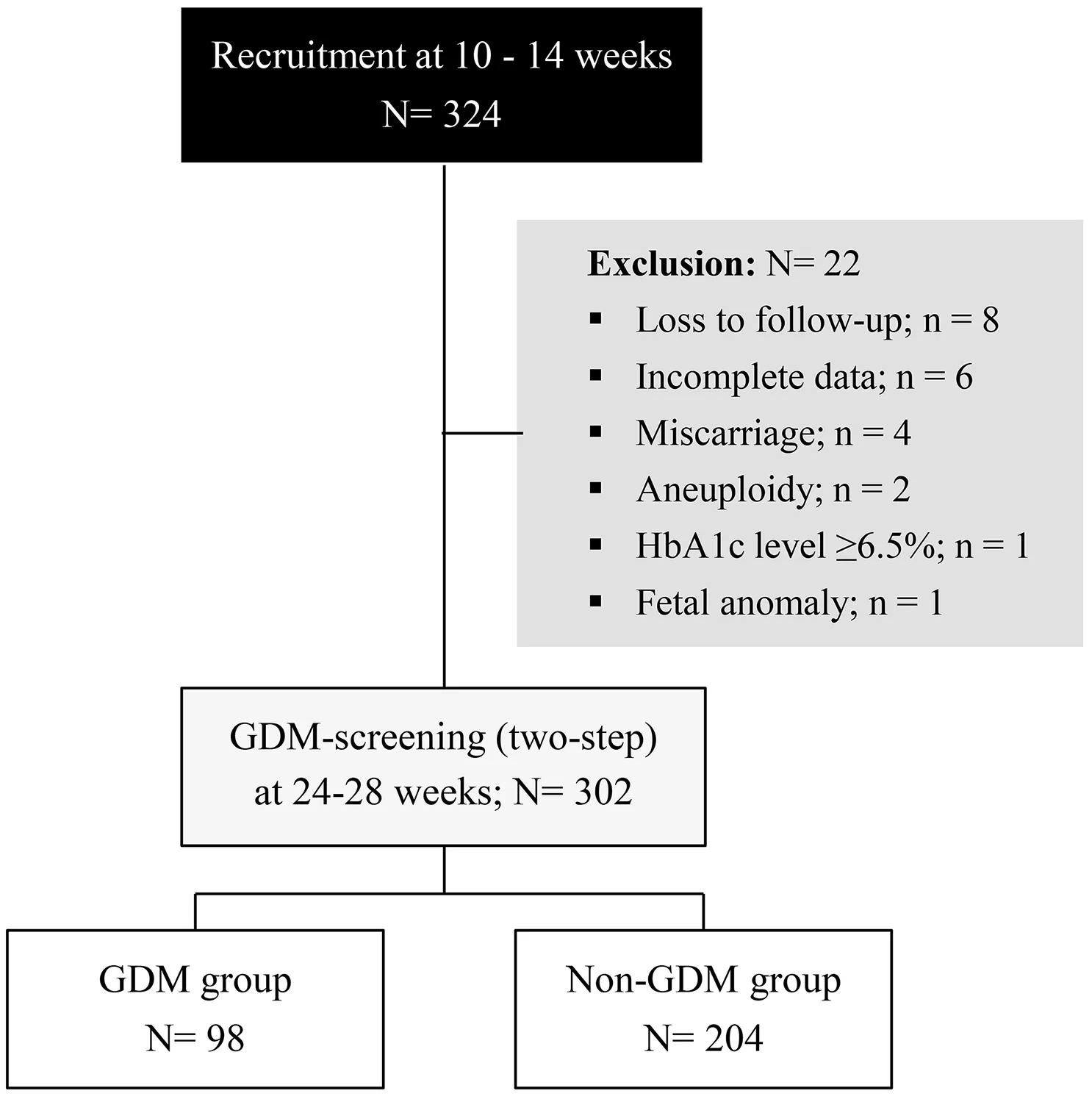
Flow chart of the study protocol. GDM: gestational diabetes mellitus.

### Reference ranges of first trimester asprosin

Between 10 and 14 weeks of gestation, plasma asprosin levels did not differ significantly across gestational ages, although a slight increasing trend with advancing gestational week was observed (Table 2). After adjustment for potential confounding factors using a generalized linear model, only pre-pregnancy BMI was independently and significantly associated with first-trimester asprosin levels, as presented in Table 3.

**Table 2:** Distribution of plasma asprosin levels based on gestational week.

Gestationalweek	N	Mean	SD	50^th^ percentile	25^th^ percentile	75^th^ percentile
10	22	56.26	15.92	56.55	45.34	65.42
11	45	57.64	23.00	55.18	42.07	70.67
12	89	59.08	21.67	54.56	43.95	71.61
13	119	60.55	17.26	59.99	47.93	70.47
14	27	61.91	19.93	65.16	45.49	73.03

SD, standard deviation.

**Table 3:** Generalized linear model analysis exploring factors associated with asprosin levels.

	Coef.	Std. Err.	P-value	95% CI	
Pre-pregnancy BMI	1.193	0.237	<0.001	0.728	1.658
Maternal age (year)	−0.144	0.237	0.544	−0.609	0.321
Family history of DM	−3.449	3.048	0.258	−9.423	2.525
Dyslipidemia	0.774	9.635	0.936	−18.111	19.659
Gestational age (week)	0.621	1.161	0.593	−1.654	2.896
Parity (ref. = 0)	3.728	2.378	0.117	−0.932	8.389
Constant	27.362	17.043	0.108	−6.042	60.766

Coef., coefficient; Std. Err., standard error; BMI, body mass index; DM, diabetes mellitus; CI, confidence interval; ref., reference.

Regression analysis of the relationship between plasma asprosin concentrations and pre-pregnancy BMI demonstrated that a quadratic model provided the best fit, as illustrated in Fig 2. However, the strength of the association was weak, with the model explaining only 8.2% of the variability in plasma asprosin concentrations (R^2^ = 0.082), despite reaching statistical significance (p < 0.001). The resulting regression equation was as follows:

**Fig 2:**
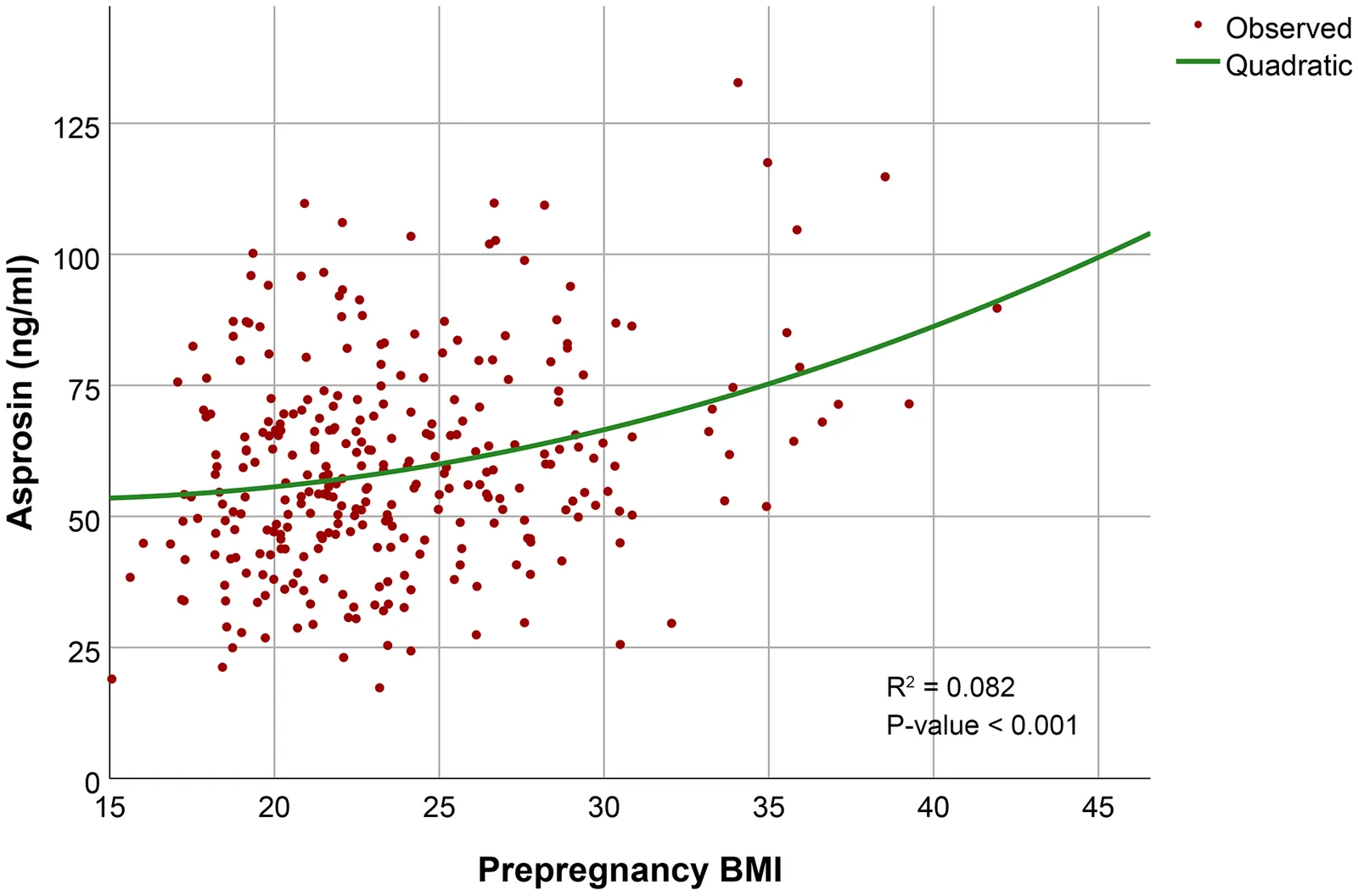
Asprosin levels as a function of maternal pre-pregnancy BMI (kg/m^2^) (BMI, body mass index.).

Predicted mean asprosin (ng/mL) = 60.298 - 1.115 × BMI (kg/m^2^) + 0.044 × BMI^2^ (kg/m^2^)

(p < 0.001; R^2^ = 0.082)

Based on the predictive model, the absolute residual was not significantly correlated with pre-pregnancy BMI (p = 0.558). The predicted SD remained constant across the range of pre-pregnancy BMI at 16.00 ng/mL.

### Associations of first trimester asprosin and subsequent development of GDM

First-trimester plasma asprosin concentrations were comparable between women who subsequently developed GDM and those who did not, as illustrated in Fig 3. The mean asprosin level was 59.4 ± 20.0 ng/mL in the GDM group and 59.7 ± 19.0 ng/mL in the non-GDM group.

**Fig 3:**
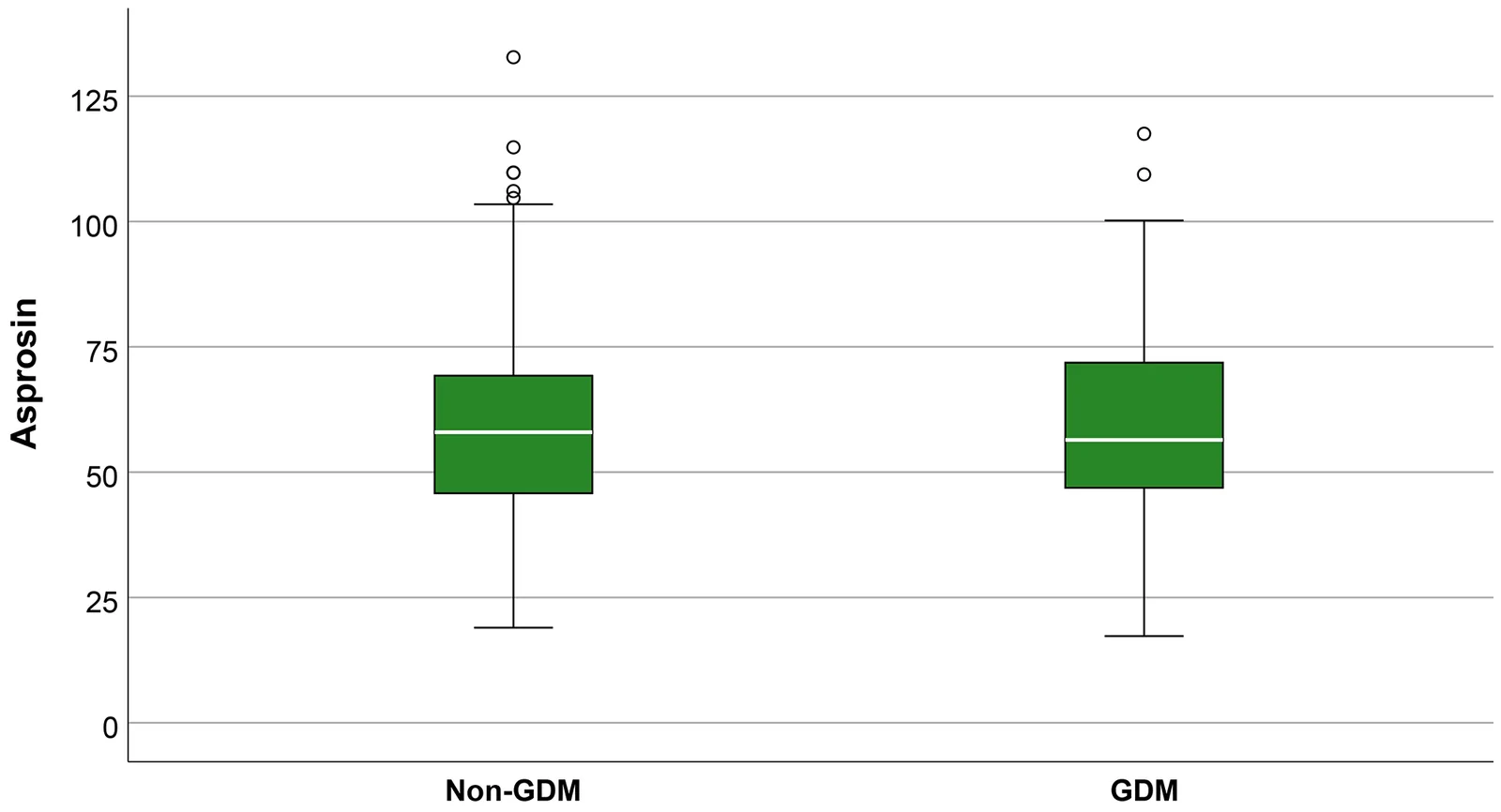
Boxplots for comparison of asprosin levels between the two groups (GDM, gestational diabetes mellitus).

On multivariable logistic regression analysis, first-trimester asprosin levels were not independently associated with the subsequent development of GDM after adjustment for potential confounding factors, as shown in Table 4. The model demonstrated a good fit, as indicated by a non-significant Hosmer-Lemeshow goodness-of-fit test (p = 0.837). In contrast, maternal age and pre-pregnancy BMI remained significant independent predictors of GDM. ROC curve analysis was performed to evaluate predictive performance (Fig 4). Maternal age and BMI demonstrated modest but statistically significant discriminatory ability for GDM. However, first-trimester asprosin exhibited no significant predictive value, with an area under the curve (AUC) of 0.516 (95% CI: 0.447 to 0.585), as presented in Table 5.

**Table 4:** Predictive logistic regression model of first-trimester risk factors for gestational diabetes mellitus.

Risk factors	Coefficient	Standard error	z	P-value	95% CI
Asprosin	−0.005	0.007	−0.67	0.503	−0.019 to 0.009
Maternal age	0.122	0.031	4.00	0.000	0.062 to 0.182
Pre-pregnancy BMI	0.096	0.031	3.12	0.002	0.036 to 0.157
Parity	−0.466	0.295	−1.58	0.114	−1.044 to 0.111
Family history of DM	0.853	0.347	2.46	0.014	0.172 to 1.534
Chronic hypertension	0.341	1.023	0.33	0.739	−1.664 to 2.346
History of GDM	1.109	1.012	1.10	0.273	−0.874 to 3.092
Gestational age	0.109	0.142	0.77	0.442	−0.169 to 0.387
constant	−7.985	2.187	−3.65	0.000	−12.272 to −3.699

CI, confidence interval; BMI, body mass index; GDM, gestational diabetes mellitus.

**Table 5:** Areas under the ROC curves of individual first-trimester risk factors and the predictive model for GDM.

Risk factors	Areas under the ROC curve	95% CI
Asprosin	0.516	0.447 to 0.585
Maternal age	0.624	0.558 to 0.690
Pre-pregnancy BMI	0.617	0.549 to 0.684
Family history of DM	0.561	0.513 to 0.608
Dyslipidemia	0.500	0.500 to 0.500

ROC, receiver operating characteristic; CI, confidence interval; BMI, daibete index; DM, diabetes mellitus.

**Fig 4:**
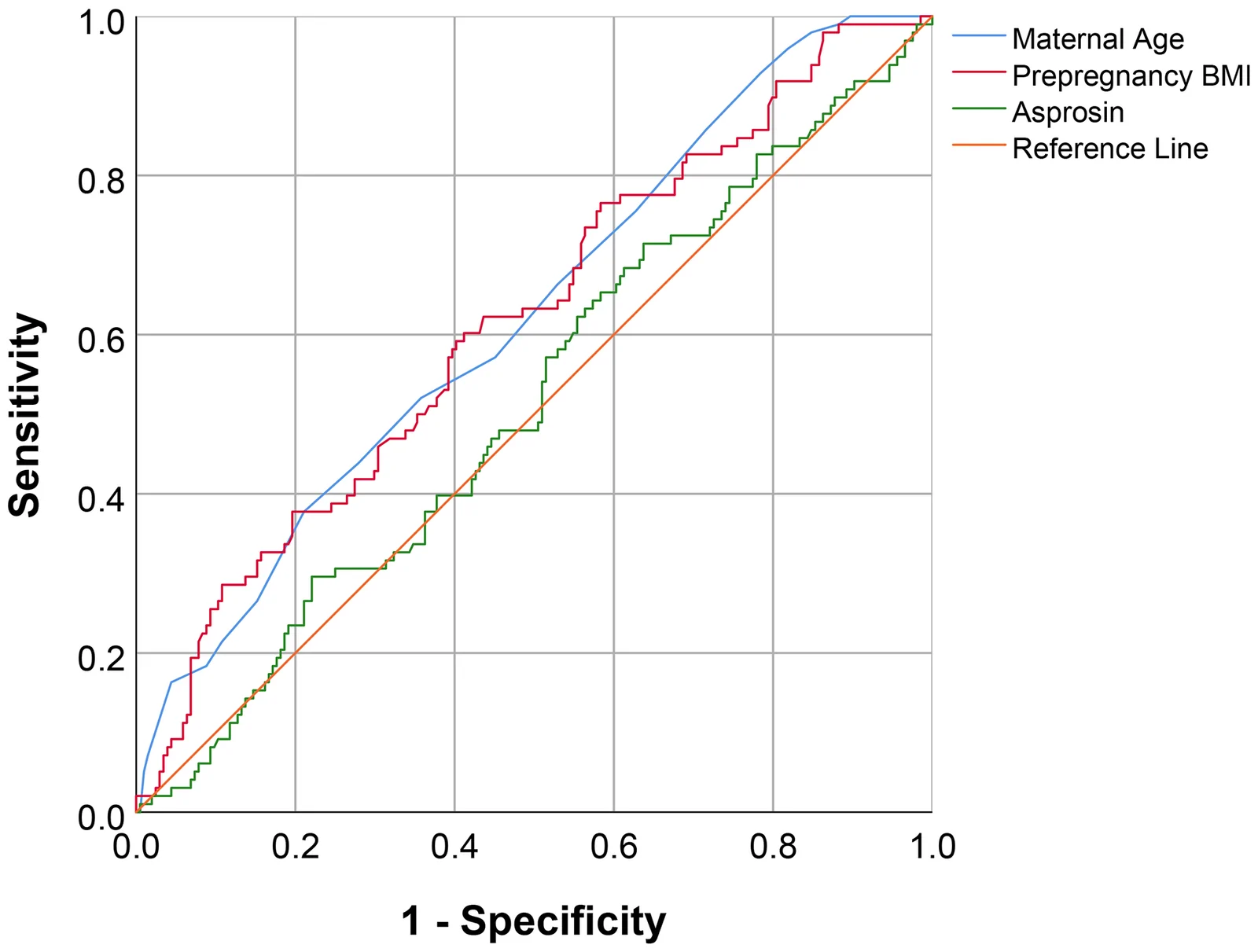
ROC curves for maternal age, pre-pregnancy BMI and asprosin in predicting GDM. (ROC, receiver operating characteristic; BMI, body mass index).

## Discussion

The principal findings of this study are as follows: 1) among low-risk pregnant women, first-trimester asprosin levels were significantly correlated with pre-pregnancy BMI; 2) A normal reference range for first-trimester asprosin concentrations was established, and a correction equation adjusted for pre-pregnancy BMI was developed. Notably, gestational age within the first trimester did not have a significant impact on circulating asprosin levels; and 3) first-trimester asprosin levels did not discriminate between women who subsequently developed GDM and those who did not, despite the high predictive performance reported for mid-pregnancy assessments in previous studies. 4) The prevalence of GDM was very high in our study (32.5%). It has long been recognized that the prevalence of GDM is relatively high among Thai women, particularly over the past two decades. This trend is likely attributable, at least in part, to the increasing proportion of pregnancies among women of advanced maternal age, which reached 22% in 2022 [12], and has continued to rise in recent years. The increasing prevalence of overweight women in reproductive age may also have contributed to this trend. It should be emphasized that the low-risk cohort in the present study did not exclude women of advanced maternal age or those with obesity. Furthermore, although our institution is a tertiary referral center, the data analyzed in this study were derived from routine GDM screening of consecutively recruited low-risk pregnant women attending our antenatal care clinic, rather than from high-risk cases referred from other hospitals, thereby minimizing the potential for selection bias. In general, GDM itself is not a common indication for referral to our center. Therefore, the high prevalence of GDM observed in this study is likely to be representative of that in the general Thai pregnant population.

We observed that the normal plasma asprosin concentrations in this cohort were relatively higher than those reported in previous studies involving Western populations. Ethnic differences may partly account for this discrepancy. Normally, asprosin is primarily released by white adipose tissue; however, during pregnancy, the placenta contributes to maternal circulating asprosin levels [13], and although the absolute amount secreted may be comparable across populations, Thai women generally have a smaller body habitus and lower total blood volume [14,15]. This may result in relatively higher measured concentrations of placentally derived asprosin. Furthermore, our finding that first-trimester plasma asprosin increases with maternal pre-pregnancy BMI is consistent with recent evidence demonstrating a strong positive correlation between maternal BMI and placental asprosin expression [13]. Accordingly, in addition to maternal BMI, ethnic background should be considered when interpreting circulating asprosin levels.

Circulating asprosin has been reported to be pathologically elevated in insulin-resistant states, including obesity and type 2 DM [16]. In pregnancy, Baykus et al. [17] demonstrated higher maternal and umbilical cord blood asprosin levels in women with GDM compared with controls. Similarly, Zhong et al. [10] reported increased plasma asprosin concentrations at 18–20 weeks’ gestation in women who later developed GDM, and Boz et al. [11] showed high diagnostic performance of mid-pregnancy asprosin for GDM detection. The discrepancy between those findings and our results may be attributable to differences in gestational timing. Although second-trimester asprosin concentrations were not measured in the present study, the contradictory findings between our first-trimester results and those reported in previous second-trimester studies suggest that the discriminatory performance of asprosin may be gestational age-dependent. These contrasting observations may be explained by two hypotheses. First, it is well established that women who subsequently develop GDM exhibit subtle or subclinical insulin resistance in early pregnancy, which becomes clinically apparent later in gestation as pregnancy-induced insulin resistance progressively increases [18]. Accordingly, our findings indirectly suggest that asprosin may not adequately reflect subclinical insulin resistance and, therefore, may have limited ability to predict the subsequent development of GDM when measured in early pregnancy. Second, elevated asprosin concentrations may represent a consequence rather than a precursor of GDM, increasing only after the metabolic disturbances associated with the disorder have become established. In other words, circulating asprosin levels may be dependent on the severity of the diabetic state or the degree of metabolic derangement, becoming abnormal only after GDM has clinically developed. Consequently, asprosin appears to have limited utility as a long-term predictive biomarker for GDM, particularly when assessed in early gestation, well before the onset of overt disease. Note that, we did not measure asprosin levels during the second trimester because previous studies have consistently demonstrated higher asprosin levels in women with GDM than in those without GDM during this period. Moreover, the primary objective of the present study was to determine whether first-trimester asprosin concentrations could serve as an early predictor of subsequent GDM development. Nevertheless, the absence of second-trimester asprosin measurements represents an important limitation of this study, as it precluded the direct assessment of longitudinal changes in asprosin concentrations throughout gestation and prevented comparisons of temporal patterns between women who subsequently developed GDM and those who did not.

**Strengths and limitations:** The strengths of this study include its high reliability, supported by the prospective design, consecutive recruitment that minimizes selection bias, an adequate sample size or power to address the main objective with high reliability, and a homogeneous cohort regarding baseline metabolic health. The limitations include the single-center design in a Thai population, which limits generalizability, underscoring the need for external validation in diverse settings, and absence of asprosin measurements at the time of GDM diagnosis, thereby precluding assessment of its discriminatory performance at 24–28 weeks.

**Clinical Implication:** Asprosin does not appear to be a useful biomarker for first-trimester screening of GDM. Nevertheless, the first-trimester normative data established in this study may serve as a reference for future research and clinical interpretation, although ethnic variability should be taken into account.

**Research implication:** Although the present findings do not support the clinical utility of asprosin as a first-trimester screening marker for GDM, its favorable performance in differentiating GDM from non-GDM in late pregnancy indicates potential clinical relevance. Accordingly, further investigations are warranted to determine whether first-trimester asprosin may be effective in screening for pregestational diabetes mellitus, particularly in comparison with HbA1c, given that pregestational diabetes is characterized by preexisting insulin resistance early in gestation. In addition, future studies should explore its potential role in other clinical applications, such as predicting the subsequent need for insulin therapy.

**Conclusions:** A normal reference range for first-trimester asprosin concentrations has been established and may prove valuable for future clinical and research applications. In interpreting asprosin levels, both maternal BMI and ethnicity should be carefully considered. However, first-trimester asprosin does not appear to be an effective predictor of GDM and offers limited clinical utility for risk stratification of subsequent GDM.

## Supporting information

S1 File Asprosin GDM data PLOS One. (XLSX)
